# Diabetes

**Published:** 1867-09

**Authors:** 


### DIABETES.

The plan Dr. Hare, of University College Hospital, adopts in cases of diabetes, is to allow the patients, when first admitted, the diet of the hospital in as great quantities as they desire. After pursuing this plan for a day or two, he changes to the usual restricted diet for diabetic patients, and can thus ascertain for himself the exact difference so produced. The effect is quiet amazing. The quantity of urine is diminished, as is the amount of sugar contained in it, and by persevering in this plan of dieting he is able to reduce the specific gravity of the urine to a very low standard. In one case recently under his care it came down to 1007, yet there was a trace of sugar present. Cases have been recorded where sugar was found in urine having a specific gravity of 1015 or 1016, but we believe that this is one of the, if not the very, lowest densities presented by urine containing sugar. Dr. Hare usually combines his dietetic treatment with the exhibition of the tinct. ferri perchloridi and opiates night and morning.—*Medical Times and Gazette, January 12, 1867.*

